# Central Ameloblastoma with a Peripheral Ameloblastoma-Like Component: A Case Report

**Published:** 2014-09

**Authors:** Seyyed Hosein Tabatabaei, Mohammad Hassan Akhavan Karbasi, Mohammad Danesh Ardekani, Neda Gholami, Arezo Khabazian

**Affiliations:** 1 Department of Oral and Maxillofacial Pathology, Faculty of Dentistry, Shahid Sadoughi University of Medical Sciences, Yazd, Iran;; 2 Department of Oral and Maxillofacial Medicine, Faculty of Dentistry, Shahid Sadouhji University of Medical Science, Yazd, Iran;; 3 Department of Oral and Maxillofacial Medicine, Zanjan University of Medical Science, Zanjan, Iran;; 4 Department of Periodontology, Faculty of Dentistry, Shahid Sadoughi University of Medical Sciences, Yazd, Iran

**Keywords:** Ameloblastoma, Mandible, Iran

## Abstract

Amebloblastoma as the most common epithelial odontogenic neoplasm may occur in two forms of central and peripheral. This report presents a case of a 41-year-old Iranian female with a six-month complaint from a painless mass in the right posterior portion of the mandible. The case was diagnosed as an exophytic epulis-like peripheral component with characteristics of peripheral ameloblastoma and an intrabony component like solid ameloblastoma. Two probable hypotheses considered for this case is also discussed.

## Introduction

As the most common epithelial odontogenic neoplasm,^1^^,^^2^ ameloblastoma may arise from dental lamina, developing enamel organ, epithelial lining of an odontogenic cyst or basal cells of oral mucosa. It is noted that ameloblastoma mainly occurs in jaw bones. Intraosseous cases can be seen in two forms of solid (multicystic) and unicystic.^2^ They are slow growing and locally invasive tumors that in most instances run a benign course.^2^^,^^3^ Solid (multicystic) ameloblastoma usually invades the bone marrow spaces^4^ and causes bone expansion without perforation. Peripheral ameloblastoma is an uncommon variant of ameloblastma,^2^^,^^5^ that comprises about 1-10% of all ameloblastomas.^2^ This type of ameloblastoma cannot extend beyond the gingival mucosa into the alveolar bone.^6^ Although in some cases larger tumors may cause mild saucerization of adjacent bone, but bone involvement usually is not significant.

This article reports clinical, radiographic and microscopic features of a rare case of central solid ameloblastoma. It caused an exophytic gingival mass with characteristic microscopic features of a peripheral ameloblastoma through perforation of cortical alveolar ridge. A literature review with specific focus on its pathogenesis and origin is also presented. In preparation of this case report, patient’s consent was obtained.

## Case Report

A 41-year-old Iranian female was referred to Yazd Faculty of Dentistry with six-month complaint from a painless mass in the right posterior portion of the mandible. According to the patient, the soft tissue mass enlarged gradually during the six-month time period. Her medical history was normal and she was in good general health condition.

**Figure 1:**
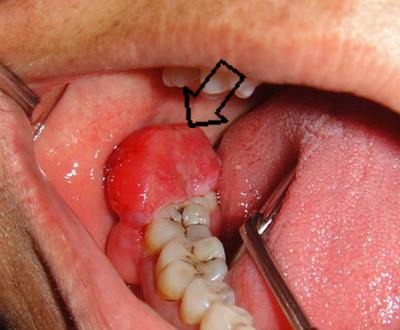
Clinical view: a firm pedunculated gingival mass in the posterior region of the right third molar tooth with anteriorly and buccally extension (black arrow).

Intraoral examination revealed a firm pedunculated gingival mass which was 3×3.5 cm in extent, located in the posterior region of the right third molar tooth, extending anteriorly and buccally and covering crown of the tooth. The lesion was lobulated and covered mainly with a normal colored mucosa (figure 1). Slight expansion of buccal and lingual cortex of the third molar region could be seen. These plates were firm and nontender in palpation. The molar teeth did not have any restoration, caries or significant loosening. No asymmetry was obvious in extra oral examinations.

**Figure 2:**
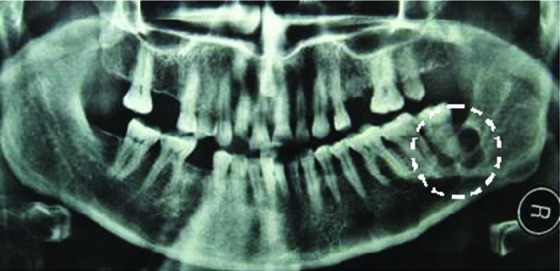
Panoramic view: a soap bubble multilocular radiolucency with well-defined margins in mesial, distal and periapical of third molar (white circle).

**Figure 3:**
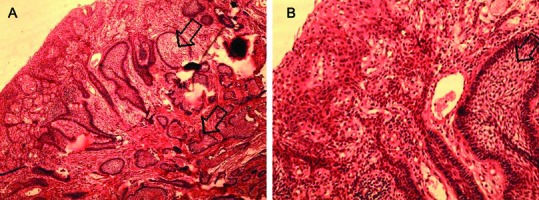
Incisional biopsy of gingival mass, (A): the lamina properia  shows multiple  ameloblastomatous islands with various sizes (black arrow) and shapes in a background of a fibrous stroma. (B): Fusion of tumor islands with basal layer of epithelium parts (white circles), (×10 magnification).

**Figure 4:**
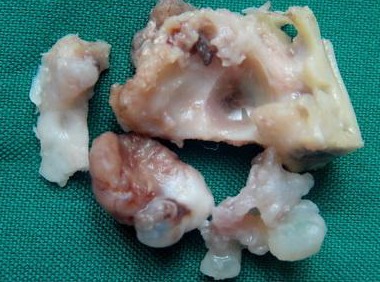
Gross view of surgically marginal resected specimen.

Differential diagnosis of gingival exophytic lesions in retromolar pad area along with fibrous nodules, pyogenic granuloma, peripheral giant cell granuloma, peripheral ossifying fibroma and other peripheral hyperplastic masses were considered.^2^^,^^3^ In panoramic view, a soap bubble multilocular radiolucency with well-defined margins was apparent. It measured approximately 3×3.5 cm in mesial, distal and periapical of third molar (figure 2). Incisional biopsy of gingival mass, extraction of third molar and curettage of intraosseous lesion was made under local anesthesia. The specimen was then sent for microscopic examination. Microscopically, hematoxylin and eosin stained section of soft tissue component revealed a stratified squamous epithelium. The lamina properia showed multiple islands with various sizes and shapes in a background of fibrous stroma with few blood vessels. These islands were lined peripherally by one layer of ameloblastic columnar cells. The islands contained stellate reticulum-like cells within them. Fusion of tumor islands with basal layer of epithelium was evident in some parts. Microscopic evaluation of curetted specimen (figures 3A and 3B) also revealed multiple island of follicular ameloblastoma in a mature fibrous stroma. Due to the diagnosis of follicular ameloblastoma, marginal resection was performed and the lesion resected completely (figure 4). Finally, on the basis of microscopic and clinicoradiographic findings, the present case was diagnosed as solid ameloblastoma (follicular type) with a peripheral ameloblastoma- like component.

## Discussion

Ameloblastoma, most commonly present itself as an intraosseous lesion and rarely as a peripheral lesion in gingival or alveolar mucosa.^2^ This case report consists of an exophytic epulis-like peripheral component in retromolar region (figure 1) with characteristics of peripheral ameloblastoma, and an intraosseous component (figures 2 and 3) like solid ameloblastoma that caused a well-defined multilocular radiolucency in mesial, distal and periapical portion of the third molar and expansion of buccal and lingual plates.

Microscopic evaluation of mucosal component revealed an ameloblastoma like structure as was seen in central part that partially was attached to the basal layer of oral mucosa (figure 3). Based on clinical, radiographic and microscopic indications, two probable pathogeneses that could be considered for this lesion were:

1- A peripheral ameloblastom with an intraosseous infiltration

2- A central ameloblastoma with extention to the soft tissue

With respect to the first probable pathogenesis, proposed diagnostic criteria for peripheral ameloblastom ^7^are:

1) Originating from the overlying epithelium

2) Presence of odontogenic epithelial islands in the lesion

3) Lack of a potential to bone infiltration

In the present case, according to the microscopic view of exophytic portion, ameloblastomatous structures was seen with follicular pattern that was attached to basal layer of the oral mucosa. These characteristics were in favor of peripheral ameloblastoma. However, most oral surgeons and pathologists consider the peripheral ameloblastoma as a non-invasive lesion which cannot penetrate into the underlying bone.^8^^,^^9^ Although few cases of peripheral ameloblastoma with jawbone resorption is also reported,^6^^,^^8^^,^^10^^,^^11^ but this type of resorpsion is due to tumor pressure rather than its invasion. Akio uedo^11^(1998) has reported a peripheral ameloblastoma with unusual invasive features and invasion of maxillary posterior region. This scenario finally led to death of the patient (after 8 years and three recurrences) as it interfered with vital structures and penumonia. Even if one considers the possible occurrence of peripheral ameloblastoma with invasive characteristics, the short presence (6 months) of gingival exophytic lesion in the present case could not lead to bone resorption.

Therefore, it appears that the first hypothesis about pathogenesis of this lesion is less likely. In the present case, tumor connection to oral mucosa (figure 3B) seems to indicate that the tumor originated from basal layer of the oral mucosa,^2^ but like few other samples, it only can be due to simple connection of proliferating tumor cells to basal layer of the epithelium

Second probable pathogenesis: central ameloblastoma is most likely to expand the bone rather than its perforation.^5^ In some patients, intra osseous ameloblastomas being left without treatment may lead to bone expansion or grotesque portions, but usually they do not perforate the bone. For unknown reasons, some central ameloblastomas as in the present case perforate the bone and extend to the adjacent soft tissues.^4^ In few articles^1^ the report of the first peripheral ameloblastoma has been accredited to Kuru in 1911.However, the mentioned lesion was an intraosseous ameloblastoma that perforated the alveolar bone, attached to the epithelium of oral mucosa and finally was appeared clinically as a peripheral lesion.^8^^,^^12^ Similar cases are reported by Tongdee and Gangykavin.^13^

Ragharendra^1^ reported on a large plexiform ameloblastoma in an 11-year-old male with invasion to the soft tissue. It created an exophytic mass in anterior part of the mandible with characteristics of peripheral ameloblastoma and a large unilacular radiolucent defect with characteristics of intraosseous ameloblastoma.

Similar to the above mentioned cases, eventually the present case was diagnosed as a solid central ameloblastoma (follicular type) based on clinical, radiographic and histopathologic evidences. This originated in alveolar processes around the root of the third mandibular molar, gradually led to buccul and lingual cortical expansion and then perforated the bone and proliferated in the soft tissue. The enlarging mass subsequently integrated with the epithelial basal cells and presented as a peripheral exophytic mass with microscopic characteristics of peripheral ameloblastoma. In conclusion, although the incidence of intraosseous ameloblastoma with an exophytic mass is rare, it may have to be considered in differential diagnosis of gingival exophytic lesions in retromollar pad area along with fibrous nodules, pyogenic granuloma, peripheral giant cell granuloma, peripheral ossifying fibroma and other peripheral hyperplastic masses. In case of eventual diagnosis (after clinical, radiographic and microscopic evaluations) of an intraosseous ameloblastoma with a peripheral ameloblastoma-like component, a more extensive therapy like marginal resection rather than a conservative therapy is recommended.
